# Content analysis of chronic pain content at three undergraduate medical schools in Ontario

**DOI:** 10.1080/24740527.2017.1337467

**Published:** 2017-08-04

**Authors:** Leigha Comer

**Affiliations:** York University, Toronto, Ontario, Canada

**Keywords:** education, curriculum, chronic pain

## Abstract

**Background**: It has been well documented that interdisciplinary, comprehensive pain education can foster positive pain beliefs among medical students, in addition to improving students’ abilities to diagnose and treat pain. Though some work has been done to quantify the number of hours of pain education students receive, the content itself has received little attention.

**Aims**: This study seeks to identify what medical students learn about chronic pain throughout an undergraduate medical degree program in Ontario.

**Methods**: Three undergraduate medical schools in Ontario were selected on the basis of variety in curricular structure and instructional methods. Written documents comprising the formal curriculum were analyzed through qualitative and quantitative content analysis. These findings were compared with promising practices from the pain education literature.

**Results**: The three curricula studied here dedicate the bulk of pain education to three topics: pain mechanisms, pain management, and opioids and addiction. The curricula vary considerably in organization of content and hours of pain training. All three curricula were found to contain negative pain beliefs that characterize pain patients as difficult, overwhelming, and unrewarding to work with. Two of the medical schools studied here do not have a pain curriculum.

**Conclusions**: The results of this study indicate a need for medical schools to develop comprehensive, interdisciplinary pain curricula. Though increasing the number of hours of pain training is crucial, equally imperative is a consideration of what, and how, students learn about pain.

## RÉSUMÉ

**Contexte**: Il est bien documenté que l’enseignement interdisciplinaire et global de la douleur en médecine peut favoriser une modification positive à l’égard des croyances positives chez les étudiants en médecine, en plus d’augmenter leur capacité à diagnostiquer et à traiter la douleur. Bien que certains travaux aient été menés pour quantifier le nombre d’heures prodiguées en éducation sur la douleur le contenu des cursus a reçu peu d’attention.

**But**: Cette étude cherche à déterminer ce que les étudiants en médecine dans la province de l’Ontario apprennent au sujet de la douleur chronique tout au long de leurs études.

**Méthodes**: Trois curriculums de médecine de premier cycle de l’Ontario ont été choisies de manière à refléter la diversité des structures et des méthodes d’enseignements. Les documents écrits comprenant le programme – plan cours officiel ont été scutés par une analyse de contenu qualitative et quantitative. Les observations ont été comparées à des pratiques prometteuses décrites dans la littérature sur l’éducation médicale de la douleur.

**Résultats**: Les trois curriculums étudiés consacrent la majeure partie de l’éducation à la douleur à trois sujets: les mécanismes de la douleur; la gestion de la douleur; et les opioïdes et la dépendance. Le contenu des curriculum varient considérablement en ce qui concerne le cursus de formation et le nombre d’heures qui y sont consacrées. Les trois curriculums contenaient des croyances négatives sur la douleur décrivant les patients souffrant de douleur comme étant des sujets difficiles, exigeants et avec lesquels il est peu gratifiant de travailler.

**Conclusions**: Les résultats de cette étude révèlent la nécessité pour les Facultés de médecine d’élaborer des curriculums interdisciplinaires et globaux sur la douleur. Bien qu’il soit primordial d’augmenter le nombre d’heures de formation, il est tout aussi impératif d’examiner le contenu de ce que les étudiants apprennent au sujet de la douleur et surtout la façon dont ils l’apprennent.

## Introduction

Writing in 1983, Joseph Kotarba pronounced the treatment of chronic pain as one of medicine’s “greatest failures.”^1^ Medicine’s struggles to adequately address chronic pain are evident in statistics on the individual burden of living with protracted pain. In Canada, one in five adults suffers from chronic pain.^2^ These sufferers are two to three times more likely to commit suicide^3^ and experience the lowest quality of life compared to people living with other diseases such as chronic lung and heart disease.^4^ Furthermore, uncontrolled pain compromises the immune system, promotes tumor growth, and increases morbidity and mortality following surgery.^5^ It is clear that there is an urgent need for effective solutions to the puzzle of pain, particularly given the complexity of this condition.

As the gatekeepers of medicine, doctors play a key role in the diagnosis and treatment of chronic pain. Family physicians are generally the first point of contact for people suffering from chronic pain, and they are often at the center of the patient’s health care team. For these reasons, there has been significant growth in the literature on medical pain education. In 2009, for instance, Watt-Watson et al. published the results of their study of pain education in prelicensure health science programs in Canada. Their research illustrates a significant need for increased hours of pain training in medical schools across Canada.^6^ Research on pain education also suggests that medical students demonstrate a lack of knowledge about pain management^7^ and that students’ knowledge can be improved through well-designed undergraduate pain curricula.^8^ Others have identified the importance of encouraging positive pain beliefs among medical students.^9,10^

This article looks to expand on this knowledge through a content analysis of the formal, written curricula of three undergraduate medical schools in Ontario. Though the literature suggests that medical students are not receiving an adequate number of pain training hours and that the outcomes of the pain training they do receive are substandard, there has been little attention paid to what, and how, students learn about pain in an MD program. Furthermore, whereas research on curricular outcomes tends to focus on students’ knowledge and skills as the final product of medical education, this study looks to examine *what* students are learning about pain management and how this education compares to recommendations set out by bodies such as the Interprofessional Association for the Study of Pain (IASP).

## Methods

The study sample includes three of the six undergraduate medical schools in Ontario. In order to ensure anonymity, the pseudonyms school 1, school 2, and school 3 are used. The three schools studied here were chosen in order to achieve variety in location, size of program, instructional methods used, and curriculum structure. School 1 is the largest and the oldest of the three programs. School 2 is roughly half the size of school 1 and was founded almost a decade later. School 3 is the youngest and smallest of the three medical schools studied.

The written, formal curriculum of each program was collected through administrators, faculty, curriculum planners, and the deans of the programs. Documents analyzed include course syllabi, academic calendars, lists of total pain objectives, lecture slides, reading lists, and other materials. This written curriculum was supplemented by discussions with faculty in person, through e-mail, and over the telephone. Documents were analyzed with four goals in mind: first, to consider the ways in which documents comprising the formal curriculum organize institutional actions, such as the time and space given to certain topics and when this content is covered in the curriculum^11^; second, to assess pain content in relation to the literature on pain education; third, to compare between medical schools in order to explore the state of pain education across institutions; and, finally, to analyze the pain beliefs framing pain content at each medical school.

The structure and organization of each curriculum, as well as the topics covered, were compared to the recommendations outlined in the IASP Interprofessional Pain Curriculum.^12^ Differences between the three medical programs were also identified and contextualized within the wider organizational structure of the medical schools. Quantitative coding of the content itself consisted primarily of counting instances of the occurrence of certain topics and keywords, such as *opioid, gate control theory*, and *biopsychosocial*. Having counted discrete units and segments, these were then cross-tabulated to identify keywords and topics that frequently appear together. Qualitative analysis focused on the pain beliefs, pain theories, and medical models framing curricular content.^13^ Overall, this content analysis found that the three curricula studied here dedicate the bulk of pain content to the following three topics: pain mechanisms and manifestation, pain management, and opioids and addiction.

## Results

### Organization of pain content in the medical curriculum

Research suggests that contextual factors such as when pain content is integrated into the curriculum, the methods through which pain content is delivered, and the total number of hours of pain training received have an important influence on students’ pain education.^14^ Thomson, for instance, noted that medical curricula tend to include pain as a symptom of other diseases and therefore spread pain content across the curriculum in general required courses.^15^ One concern is that this approach leads to fragmentation of content, such that pain content has no “home” or discipline-specific courses; as Poyhia et al.^16^ suggest, the inclusion of pain content in general required courses throughout the curriculum risks producing an understanding of pain that is fragmented and ineffectual. Given that effective pain education requires intellectual, emotional, technical, and ethical learning, Mezei and Murinson argued that a fragmented curriculum does not provide students with the opportunity to acquire competency in each of these dimensions.^17^

Likewise, Wittert and Nelson argued for the importance of a curriculum that provides a core foundation in scientific knowledge, such that medical students can transfer information and solve problems effectively.^18^ They also suggested that though the optimal methods for content delivery have yet to be determined, case-based learning might allow for structured tutorials that provide opportunities for student participation and the development of scientific knowledge and clinical skills. Also key is an interprofessional approach that provides a common basis and shared understanding among multiple health professions. Barr et al. noted that an interprofessional medical education provides students with the opportunity to recognize the roles, responsibilities, and competencies of others and to know when, where, and how to involve other health professionals in an interprofessional health care team.^19^

Drawing on this literature, the *IASP Interprofessional Pain Curriculum* recommends that pain education take place in an interprofessional context in which core lectures provide knowledge on concepts relevant to all health professions, and small-group work allows students to focus on interprofessional patient-focused assignments.^12^ The IASP further recommends that interprofessional pain education be incorporated early in students’ education and that students learn to assess and manage pain as a multidimensional experience that requires collaboration among health professionals.

Given the importance of these structural and organizational factors in students’ pain education, this study understands the curricular documents studied here not only as reflecting what is taught in the classroom but also as texts that organize the time and space allotted to specific topics and when and how these are covered in the curriculum. To this end, the organization of pain content at each medical school was mapped with the goal of comparing this organization to the standards set out by the *IASP Interprofessional Pain Curriculum*.^12^

In contrast to the other two programs studied here, school 1 concentrates the bulk of pain content into a week focused on interprofessional pain instruction in students’ second year. Throughout this week, content is delivered through a mixture of large group lectures and small group workshops. In addition to these interprofessional sessions, pain content at school 1 is integrated into general required courses on pharmacology, neuroanatomy, and neurophysiology throughout the curriculum.

At school 2, most pain content is integrated into general required courses. Though school 2 does not have a pain curriculum, the general curriculum features over 100 pain objectives. In pre-clerkship, pain objectives are included in learning events on physiology, neuroanatomy, and clinical foundations courses. The primary pain sessions at school 2 take place during clerkship. In year 3, students learn about pain management during a session on prescribing pharmaceuticals for persistent pain. In their fourth year, students attend a lecture on chronic pain management delivered by an expert guest speaker, as well as a session on pain management organized by the anaesthesiology department.

The modular curriculum at school 3 is centered around case-based learning. Over 400 pain objectives are integrated into school 3’s curriculum, with most of these objectives met in the first 2 years of the program. Chronic pain content is mostly delivered in sessions on end-of-life care, as well as a primary pain session on physiology and pharmacology of pain taught during students’ second year. Though this session is structured as a large group lecture, the bulk of pain content at school 3 is delivered through small group sessions, structured clinical skills sessions, laboratory sessions, and community and interprofessional learning.

### Pain mechanisms and manifestation: Where are we now?

Discussions on the nature of pain perception and mechanisms can be traced back to the 17th century, when Descartes, in his *Meditations on First Philosophy*, suggested that pain is a signal that follows a direct pathway from the body to the brain.^20^ By the mid-19th century, researchers began to question how Descartes’ theory of pain accounted for variations in the perception of pain. In 1894, two competing theories of pain emerged in attempt to resolve this issue: von Frey’s specificity theory of pain and Goldscheider’s pattern theory.^21^ Whereas the specificity theory posits the existence of specific pain receptors that transmit signals to a pain center in the brain, the pattern theory suggests that the brain receives pain messages when stimuli combine to produce a pattern or combination of pain signals.

The specificity theory of pain came to dominate medical practice and thought, due largely to its adherence to conventional biomedical assumptions about the existence of one cause for one symptom.^1^ In 1965, Melzack and Wall proposed the gate control theory of pain in an article published in *Science*.^22^ In this paper and their subsequent book, *The Challenge of Pain*, the authors highlighted the role of a “gate” in the dorsal horn that modulates the transmission of sensory information to transmission cells in the spinal cord.^23^ The gate control theory offers a physiological explanation for the role of psychosocial and other factors that mediate the transmission of pain signals, while also providing a neural basis for the phenomenon of pain. Though eventually accepted by researchers as the dominant pain theory, Kotarba noted that the gate control theory was not readily incorporated into medical curricula.^1^ At the time of writing, for instance, he reported that most medical schools continued to teach the specificity theory, despite agreement in the literature that the gate control theory of pain more effectively explains the mechanisms underlying the perception of pain.^1^

A clear, consistent understanding of pain mechanisms is vital for making sense of how patients come to experience pain and how their discomfort might be alleviated. As such, it is no surprise that the medical programs studied here devote a significant amount of space in their curricula to content on pain mechanisms. It is also encouraging to note that the three medical schools studied here teach the gate control theory of pain as the dominant pain theory. In the primary session on pain mechanisms at school 1, for instance, the summary of the major pain theories highlights gate control theory as the most comprehensive and accurate. Though no explicit mention of the gate control theory could be found in the formal curriculum at school 2, the bulk of content on pain mechanisms includes key aspects of the gate control theory, including descriptions of ascending and descending modulation, as well as the role of the dorsal horn in conditions such as central sensitization. Meanwhile, school 3 includes the gate control theory of pain in numerous objectives and in course content in pain-specific and general required courses, with no mention of the specificity theory.

This move toward teaching the gate control theory as the dominant—or only—pain theory shifts the conceptualization of pain as simply a matter of nociception and instead toward an understanding of pain as a multidimensional experience. Though this is a positive step forward, a critical analysis of curricular content on pain mechanisms reveals that essential aspects of the gate control theory tend to be minimized or unstated, particularly those that challenge orthodox biomedical thought. In developing the gate control theory of pain, Melzack and Wall were concerned primarily with the puzzle of pain: why pain and injury are not always related and what activities of the nervous system intervene between injury and pain perception such that this relationship is highly variable.^23^ One of the ways in which the authors solve this puzzle of pain is to emphasize psychosocial dimensions of pain mechanisms.

In order to understand the significance of the gate control theory, it is therefore vital to contextualize the development of this theory in light of the authors’ desire to account for these psychosocial dimensions of pain. To this end, the *IASP Interprofessional Pain Curriculum* includes the “historical development of pain theories and basis for current understanding of pain” as part of its recommended pain curriculum.^12^ Despite the importance of this history, only school 1 includes any content on the historical development of the gate control theory. Likewise, the IASP also lists a number of topics related to pain mechanisms that health professionals are expected to know, such as “the multiple dimensions of pain [including] physiological, sensory, affective, cognitive, behavioural, social/cultural/political.”^12^ Content on pain mechanisms at all three medical programs studied here, however, includes little to no mention of these aspects of pain mechanisms. Instead, content focuses almost exclusively on the physiological dimensions of pain.

The gate control theory changed our understanding of pain mechanisms by investigating the variable relationship between pain and injury. By divorcing the gate control theory from the context in which it was developed and the authors’ concern that pain be understood as a unified stream of experience, the underlying framework of the theory and the critical lens through which it conceptualizes pain mechanisms are ignored. The gate control theory of pain requires an understanding of pain within the context of a wholly integrated nervous system in which psychosocial factors are not merely secondary considerations but central to every stage of the process. These limitations suggest that in many ways the curricula studied here pay lip service to the gate control theory by highlighting it as the most accurate pain theory and then discounting many of its primary concerns. In doing so, the bulk of curricular content focuses on pain mechanisms in what Gatchel calls “purely physiological terms,” (ref. 24, p. 7) painting an incomplete picture for medical students who subsequently learn to diagnose and treat chronic pain on the basis of this understanding of pain mechanisms.

### Pain management and pharmacology of pain

The bulk of pain content in each curriculum is dedicated to content on pain management. Reflecting contemporary trends in pain management, the three medical curricula studied here emphasize pharmaceuticals as the treatment of choice for acute and chronic pain. In each of the curricula, *pain management* is synonymous with *pharmacology of pain*, because very little content in these sessions is dedicated to other treatment modalities. Sessions on pain management at each program are typically divided into three topics: the mechanisms of action underlying analgesics, guidelines for prescribing and dosing analgesics, and guidelines for prescribing opioids and screening for addiction. Though each curriculum allows some space for content related to nonpharmaceutical pain treatments and complementary and alternative (CAM) therapies, these are generally condensed into a single objective.

At school 1, most of the content on pharmacology of pain is taught during the interprofessional pain-specific sessions organized in students’ second year, including in particular a session organized by the university’s faculty of pharmacy. Of interest is the fact that school 1 does not include any sessions focused more broadly on pain management or pain treatment, instead combining all aspects of pain management into a single lecture on pharmacology. Students at school 1 also attend a lecture on the use of cannabinoids for pain, and they learn about points of intervention for pharmaceuticals in the primary session on pain mechanisms.

At school 2, most of the content on pain management focuses almost exclusively on pharmaceuticals. One exception is the expert guest lecture in students’ fourth year, which describes the ideal treatment for chronic pain as one that encompasses physical and rehabilitative therapy, as well as psychological support. In contrast, the other primary sessions on pain management at school 2 view pain management through the lens of pharmacology. For instance, whereas a session on multimodal analgesia presents an opportunity to describe a biopsychosocial, interprofessional approach to pain management, instead this session is limited to a discussion of pharmaceuticals and issues of dosing and prescribing.

The primary large group lecture on pain management at school 3 places significant emphasis on pharmacology, with nine objectives related to the pharmacological treatment of pain and a single objective pertaining to nonpharmacological pain treatments. This single objective on alternative treatment modalities focuses on physical therapies and surgical interventions, with no mention of psychosocial or other CAM treatments. Though other general required courses at school 3 do include content on nonpharmacological treatments for pain, these are primarily taught during sessions on end-of-life care.

### Opioid analgesics in the treatment of chronic non-cancer pain

The majority of content on pain management at all three programs focuses on opioid analgesics. The use of opioids in the treatment of chronic non-cancer pain is controversial due not only to issues of adverse effects and safety^25^ but also concerns that dependence, tolerance, and addiction can arise from prolonged use.^26^ In contrast, others argue that opioids are underutilized in clinical practice due to physicians’ opiophobia, such that chronic pain is often left undertreated or untreated.^2^ Given that opioid use for chronic pain is heavily contested, curricular content related to opioids has been the object of considerable scrutiny.

The *IASP Interprofessional Pain Curriculum*^12^ includes only one objective related to opioids, three objectives related to addiction, and two objectives on substance abuse. In contrast, the three medical schools studied here include a significant amount of content related to opioids, substance abuse, and addiction in sessions on pain management. School 1, for instance, includes content on opioid medications in nearly all of its sessions: in the primary lecture on pain management, one third of the content focuses on opioids; in a subsequent session on mental health and pain, opioids are described as the “standard treatment” for pain, although students are warned in the same session that opioids “don’t work” in treating most chronic pain conditions.

At school 2, opioids and addiction are mentioned in all four of the primary sessions on pain. The clerkship preparation course on pain and prescribing in particular is organized primarily around case studies in which small groups of students must determine which opioids to prescribe to patients and how to dose these correctly. In a fourth-year course on pain management, meanwhile, opioids are described as the “drug of choice for moderate to severe pain,” and content in this session also criticizes the fact that opioids are often the only drugs ordered for chronic pain. Other challenges covered in sessions on pain management include severe pain that is unresponsive to opioids, opioid-tolerant patients, drug-related aberrant behavior, and comorbidities.

Similarly, the primary session on pain management at school 3 includes seven objectives related to opioid analgesics. Under the subtopic “pharmacological treatment” of pain, 77% of the objectives are related to opioids, with the remaining content focused on describing “non-opioid analgesics” and topical medications for the treatment of pain. In addition to this primary session, several other sessions throughout the curriculum include objectives related to opioid analgesics for pain management. Content on opioid analgesics is also included in case-based, small-group patient encounters, as well as a session on pain and addiction.

This overview of all three curricula allows some insight into the substantial amount of curricular content dedicated to opioid medications. In analyzing this content, it becomes clear that each program follows a similar pattern. Though pain management sessions at all three programs acknowledge the limits and dangers of treating chronic non-cancer pain with opioids, objectives related to opioid medications significantly outweigh curricular objectives on nonopioid medications and nonpharmacological therapies. At each school, the primary session(s) on pain cite multiple studies indicating that opioids should only be prescribed as part of a multimodal, interdisciplinary pain management plan. Having emphasized these limitations, each session then proceeds to dedicate most of the content to discussing opioids.

The abundance of opioid-related content in the curriculum is concerning not only due to the quantity of time spent discussing these topics at the expense of other pain management modalities but also because of the language in which opioid use for chronic non-cancer pain is framed. Research on pain education and pain management suggests that students’ and physicians’ pain beliefs significantly impact the ways in which patients’ pain is treated. Pain beliefs refer to cultural or personal beliefs about pain, which might include expectations about working with pain patients, assumptions about the nature of pain, and confidence in treating patients’ pain.^27^ Hutchinson et al., for instance, have found that physicians’ preexisting assumptions regarding opioid medications influence the rates at which they prescribe opioids for chronic pain, particularly in the case of physicians who feel dissatisfied with their knowledge of opioid prescribing guidelines.^9^

Research has also demonstrated that pain beliefs can be positively shaped through medical education.^10^ Positive pain beliefs include confidence in working with pain patients, the expectation that working with these patients is rewarding and worthwhile, and the understanding that patients’ pain is real and not imaginary or the result of malingering. Positive pain beliefs are also important in treating patients’ *total pain*, which includes not only physical pain but often feelings of anger, loss of faith, fear of suffering, mental illness, and a plethora of other forms of suffering.^28^ Therefore, a balanced pain curriculum that promotes compassion and positive pain beliefs is crucial in improving the ways in which physicians manage patients’ pain.^29^

In each of the curricula studied here, the use of opioids for chronic pain is heavily linked to addiction, deviancy, and drug-seeking behavior. A cross-tabulation of keywords and themes, for instance, reveals that content on opioids is frequently linked with the keyword *addiction*. A qualitative content analysis further reveals that opioid use in treating chronic pain is couched in stigmatizing language in all three curricula. One example is the gendered nature of case studies or patient encounters featuring pain patients. At school 1, case studies on acute pain tend to focus on male patients presenting with relatively straightforward conditions such as acute rotator cuff injuries. Meanwhile, case studies focused on complex cases of fibromyalgia, chronic fatigue syndrome, and other contested diagnoses, along with challenging comorbidities such as anxiety, depression, and addiction, almost exclusively feature female patients. This pattern is repeated at schools 2 and 3.

At school 2, a significant portion of sessions on pain management are dedicated to preparing students to work with patients described as “difficult”: patients who have two or more chronically painful conditions, who might be tolerant to opioid analgesics, who are often malingerers or noncompliant, and who are “demanding” in seeking relief for their pain. In their third year, students at school 2 are presented with a number of case studies in order to prepare them to work with these difficult patients. One of these includes a woman with fibromyalgia who threatens to commit suicide if she does not receive her opioids. At the end of this session, students are asked whether they are “overwhelmed yet” at the prospect of working with pain patients. At school 3, content on opioids tends to use the term *narcotics*, a term generally avoided because it is vague and has pejorative implications.^30^ This term is used not only in a primary session on pain and pain management but also in other pain-related objectives throughout the curriculum. Across all three curricula, the use of opioids for chronic pain tends to be linked to addiction in session titles and curricular objectives.

## Discussion

This analysis of the formal curricula at three medical schools used the *IASP Interprofessional Pain Curriculum*^12^ as a resource for discovering promising practices for the development of a comprehensive pain curriculum. Though the IASP recommendations are not compulsory, the *Interprofessional Pain Curriculum* acts as an important tool for curriculum planners to develop pain content and to assess the current state of pain education. The IASP recommends that pain content be taught as part of an interprofessional curriculum that is introduced early in students’ medical education.^12^ Of the medical schools studied here, only one program (school 1) has a pain curriculum, and only school 1 has made significant efforts in ensuring that pain content is taught using an interprofessional approach. Furthermore, whereas school 1 and school 3 introduce pain content early in the curriculum, school 2 offers its primary sessions on pain in years 3 and 4.

Under the topic “Multidimensional Nature of Pain,” the IASP includes only four objectives related to pain mechanisms.^11^ The remaining objectives focus on epidemiology, the development of pain theories, and a substantial section on ethics. In contrast, the three medical schools studied here focus overwhelmingly on pain mechanisms. Furthermore, content in each curriculum tends to overlook crucial aspects of the pain experience, including what the IASP refers to as “factors influencing neurophysiology (e.g., genetics, age, sex, ethnicity).”^12^ One of the principles of the *IASP Pain Curriculum* is the recognition of pain as a sensory, emotional, cognitive, developmental, behavioral, spiritual, and cultural experience. In the discussion of pain mechanisms, however, each curriculum focuses almost exclusively on the sensory or physiological aspects of the pain experience.

In terms of pain management, the *IASP Curriculum*^12^ includes numerous objectives related to goals of pain management and pain management planning decisions. Though the primary sessions on pain management at all three medical schools include lengthy discussions of the IASP’s seventh treatment consideration—substance abuse issues, which includes only two objectives—there is little to no mention of the other six issues that the IASP recommends students take into account when choosing a treatment plan. These include political issues, health professional issues such as pain beliefs, and caregiver issues. These considerations are almost entirely absent from all three curricula. Likewise, though the *IASP Pain Curriculum* contains objectives related to pharmacological treatments for pain (of which opioids are featured only once), the IASP also highlights numerous nonpharmacological treatments such as psychological and behavioral strategies, neuromodulation, CAM, and information and communication technologies. These nonpharmacological pain treatments are largely absent from all three curricula or are condensed into a single objective in lectures on pain management.

At all three medical programs, content on opioid analgesics for the treatment of chronic pain is often framed in a manner that is stigmatizing and promotes negative pain beliefs. Given the contentious nature of treating chronic non-cancer pain with opioids, content on the use of opioid analgesics for chronic non-cancer pain must be carefully thought through. In developing this content, faculty and curriculum planners should consider not only the information students receive on prescribing guidelines but also the pain beliefs framing this content. Pain beliefs have a significant impact on doctors’ opioid prescribing practices, as well as their willingness to consider alternate treatment modalities for chronic pain.^31^ Given the evidence among doctors of negative pain beliefs regarding chronic pain patients and opioid use for chronic pain, it is particularly important that medical curricula include opportunities for students to reflect on their own perspectives regarding chronic pain and opioid medications.

There are several limitations to the present study, including most obviously a sample of only three medical schools in Ontario. However, the goal is not to generalize these findings to medical schools across Canada but instead to provide an in-depth analysis of three medical programs. The study also focuses solely on undergraduate pain education, without considering the learning that takes place in residency or other postgraduate training. Furthermore, given that this content analysis includes only the written, formal curriculum, this study does not take into account the informal curriculum (the unscripted, interpersonal teaching between faculty and students) or the hidden curriculum (the organizational and structural influences on students’ total learning experience).^32^ Future research should include the kinds of hidden and informal learning that take place in medical schools, as well as the ways in which decisions regarding curricular content are made.

The results of this study indicate the need for medical schools to develop comprehensive, interdisciplinary pain curricula. Though increasing the number of hours of pain training is crucial, equally imperative is a consideration of what, and how, students are taught about pain. A content analysis of the curricula suggests that rather than challenging negative pain beliefs among medical students, the three curricula studied here introduce and reinforce beliefs that patients are difficult and unrewarding to work with. Likewise, this analysis demonstrates a need for a more balanced curriculum at all three programs, such that the emphasis is on not solely pain mechanisms and pharmacological pain management but other core aspects of the experience of diagnosing and treating chronic pain.
